# The Transactions of the American Association

**Published:** 1853-09

**Authors:** 


*The Transactions of the American Medical Association*--Vol. V.

WE are happy to acknowledge the receipt of this valuable document.

We have already noticed Dr. Flint's Prize Essay, Dr. Clapp's Report on the Medical Botany of the United States, and have noticed at some length the proposed changes to the constitution and the grievances complained of in reference to the schools.

We may have something more to say of this in future. J.

